# Editorial Comment: Effect of a low-calorie diet on 24-hour urinary parameters of obese adults with idiopathic calcium oxalate kidney stones

**DOI:** 10.1590/S1677-5538.IBJU.2021.0140.1

**Published:** 2021-10-01

**Authors:** Sandra Barbosa da Silva

**Affiliations:** 1 Universidade do Estado do Rio de Janeiro Laboratório de Morfometria, Metabolismo e Doenças Cardiovasculares Rio de JaneiroRJ Brasil Laboratório de Morfometria, Metabolismo e Doenças Cardiovasculares. Universidade do Estado do Rio de Janeiro - UERJ, Rio de Janeiro, RJ, Brasil

## COMMENT

The present work has an interesting approach to evaluate the effect of the hypocaloric diet on the 24-hour urinary metabolic parameters of obese adults with idiopathic calcium oxalate kidney stones. Adult idiopathic calcium oxalate stone formers, with body mass index (BMI) ≥ 30 kg/m2 and a known lithogenic metabolic abnormality, were submitted to low-calorie diet for twelve weeks (1).

Obesity is a complex, multifactorial chronic disease influenced by genetic, behavioral, dietary, socioeconomic, and environmental factors (2). In addition, obesity is associated with both development of kidney disease and progression towards end stage renal failure. Some authors considered kidney stone disease (nephrolithiasis) is one of the possible factors that contribute to an increase in the burden of kidney damage carried by obesity (3). Nephrolithiasis is a common problem that can be associated with in urinary solute composition, has a multifactorial aetiology involving genetic and environmental factors. Nonetheless, the genetic influence on stone formation in the idiopathic stone remains considerable (4).

In this context, there are some modifiable risk factors for kidney stones, being obesity one these factors. Obesity is associated with insulin resistance and compensatory hyperinsulinemia, which may lead to the formation of calcium containing kidney stones, by increasing the its urinary excretion (5, 6).

Thus, weight loss can be a strategy for obese people to prevent or reduce the risk of developing kidney stones, including for those with idiopathic stones, however, some approaches are used to promote weight loss may increase kidney stone risk (7).

This way, the weight loss may improve harm management of kidney stones, depending on how it is achieved. Weight loss could be harmful to prevention of kidney stones if associated with a high animal protein diet (for example, increase the risk of uric acid stones), excessive use of laxatives or rapid loss of muscle (8, 9). It is important to note that the dietetic advice for weight loss, in this case, should be based on the type of kidney stone.

In summary, although weight loss demonstrates a good strategy in cases ofkidney stones formes, medical and nutritional follow-up is necessary in choosing the strategy to lose weight. The present work showed that the short-term modest weight loss induced by twelve weeks low-calorie diet is not associated with a decrease of 24-hour urinary lithogenic parameters in idiopathic calcium oxalate stone formers. Calcium oxalate urinary stone formation is probably multifactorial and driven by other factors than weight.
